# Selection of Suitable Reference Genes for RT-qPCR Gene Expression Analysis in Siberian Wild Rye (*Elymus sibiricus*) under Different Experimental Conditions

**DOI:** 10.3390/genes10060451

**Published:** 2019-06-13

**Authors:** Junchao Zhang, Wengang Xie, Xinxuan Yu, Zongyu Zhang, Yongqiang Zhao, Na Wang, Yanrong Wang

**Affiliations:** The State Key Laboratory of Grassland Agro-Ecosystems, Key Laboratory of Grassland Livestock Industry Innovation, Ministry of Agriculture and Rural Affairs, College of Pastoral Agriculture Science and Technology, Lanzhou University, Lanzhou 730020, China; zhangjch16@lzu.edu.cn (J.Z.); yuxx17@lzu.edu.cn (X.Y.); zhangzy17@lzu.edu.cn (Z.Z.); zhaoyq16@lzu.edu.cn (Y.Z.); wangn17@lzu.edu.cn (N.W.)

**Keywords:** *Elymus sibiricus*, reference genes, reverse transcriptase quantitative real-time polymerase chain reaction (RT-qPCR), expression stability, experimental conditions

## Abstract

*Elymus sibiricus*, which is a perennial and self-pollinated grass, is the typical species of the genus *Elymus*, which plays an important role in forage production and ecological restoration. No reports have, so far, systematically described the selection of optimal reference genes for reverse transcriptase quantitative real-time polymerase chain reaction (RT-qPCR) analysis in *E. sibiricus*. The goals of this study were to evaluate the expression stability of 13 candidate reference genes in different experimental conditions, and to determine the appropriate reference genes for gene expression analysis in *E. sibiricus*. Five methods including Delta Ct (ΔCt), BestKeeper, NormFinder, geNorm, and RefFinder were used to assess the expression stability of 13 potential reference genes. The results of the RefFinder analysis showed that *TBP2* and *HIS3* were the most stable reference genes in different genotypes. *TUA2* and *PP2A* had the most stable expression in different developmental stages. *TBP2* and *PP2A* were suitable reference genes in different tissues. Under salt stress, *ACT2* and *TBP2* were identified as the most stable reference genes. *ACT2* and *TUA2* showed the most stability under heat stress. For cold stress, *PP2A* and *ACT2* presented the highest degree of expression stability. *DNAJ* and *U2AF* were considered as the most stable reference genes under osmotic stress. The optimal reference genes were selected to investigate the expression pattern of target gene *CSLE6* in different conditions. This study provides suitable reference genes for further gene expression analysis using RT-qPCR in *E. sibiricus*.

## 1. Introduction

*Elymus* is the largest genus of the tribe Triticeae with worldwide distribution, including approximately 150 species [1]. *E. sibiricus* (Siberian wild rye), which is the typical species of the genus *Elymus*, is a perennial, allotetraploid, and self-pollination grass [2,3]. It is one of the most important forages in Northern China due to its high protein content, strong adaptability, superior cold, and drought tolerance [4]. Meanwhile, *E. sibiricus* were widely used in artificial grassland and ecological governance in recent years [5]. It is noteworthy that severe seed shattering is the main reason for seed yield losses in *E. sibiricus*. According to previous reports, the degree of seed shattering in *E. sibiricus* was related to genotypes [3,6], the development of abscission zone [4], and several key genes involved in plant hormones, lignin biosynthesis, and cell wall-degrading enzymes (e.g., *ETR*, *PAL,* and *CesA*) found by transcriptome sequencing [7]. Currently, the regulatory mechanisms associated with seed shattering in *E. sibiricus* has not been clearly elucidated. As a result, the analysis of expression patterns of these key genes will contribute to a better understanding of regulatory mechanisms regarding seed shattering in *E. sibiricus*. The appropriate reference gene is crucial for the gene expression analysis in *E. sibiricus*.

Reverse transcriptase quantitative real-time polymerase chain reaction (RT-qPCR) is a sensitive, precise, specific, rapid, and repeatable method to investigate and quantify the gene expression level [8,9,10]. However, the application of RT-qPCR are commonly affected by several factors, including the quality of RNA (purity and integrity), the efficiency of cDNA (complementary DNA) synthesis, reaction parameters, and primer specificity [11,12]. Therefore, in order to obtain the accurate normalization of RT-qPCR, it is necessary to screen the suitable reference genes for correcting the above-mentioned experimental errors [13,14].

Currently, transcriptome sequencing has been widely used in plant biological research, which greatly facilitates the understanding of important molecular mechanisms in plants [15,16]. Simultaneously, transcriptome data provide important resources for identification and exploration of reference genes. An optimal reference gene should be expressed at a constant level across various experimental conditions such as different genotypes, developmental stages or tissues, and even different abiotic stress. Hence, housekeeping genes are usually selected as the reference genes without validating their performance since their expression levels were thought to be stable [17,18,19]. Nevertheless, a large number of research studies recently found that the common reference genes like glyceraldehydes-3-phosphate dehydrogenase (*GAPDH*), β-actin (*ACT*), and 18S ribosomal RNA (*18S rRNA*) had significantly different expression levels under different tissues or different experimental treatments [20,21,22,23]. Consequently, it is essential to validate the stability of potential reference genes, which can effectively identify whether candidate reference genes are suitable for current experimental conditions. Using improper reference genes will lead to inappropriate interpretations [24]. It is reported that the expression stability of reference genes showed divergence in different species. For instance, TUB was a suitable reference gene for normalization of RT-qPCR in *Sedum alfredii* [25] and *Jatropha curcas* [26], but had the worst expression stability in *Rhododendron molle* [27] and *Camellia sinensis* [28]. The reason for this phenomenon may be the difference in the primer design and evaluation method, the distinct experimental conditions, and the diversity of gene function among different species [29]. Therefore, screening the reference genes that possess a high degree of expression stability in specific species is crucial to obtain the accurate results of RT-qPCR. Up to now, many suitable reference genes have been reported from different plants, including *Arabidopsis thaliana* [30], rice [31], Tibetan hulless barley [32], soybean [21], orchardgrass [33], Seashore paspalum [34], Kentucky bluegrass [35], and *Setaria viridis* [36]. However, there is no report for the selection of suitable reference genes for RT-qPCR analysis in *E. sibiricus*.

The objective of this study is to screen suitable reference genes with stable expression under various experimental conditions. Thirteen candidate reference genes including actin2 (*ACT2*), cyclophilin19 (*CYP19*), *DNAJ* heat shock N-terminal domain-containing protein (*DNAJ*), eukaryotic translation initiation factor 3A (*eIF-3A*), eukaryotic translation initiation factor 3C (*eIF-3C*), glyceraldehyde-3-phosphate dehydrogenase (*GAPDH*), histone 3.3 (*HIS3*), protein phosphatase *2A* (*PP2A*), TATA-binding protein 2 (*TBP2*), translation elongation factor 2 (*TEF2*), tubulin α-2 (*TUA2*), tubulin β-3 (*TUB3*), and U2 auxiliary factor (*U2AF*) were selected from the transcriptome data of *E. sibiricus* [7].

## 2. Materials and Methods

### 2.1. Plant Materials and Growth Conditions

In the present study, six wild *E. sibiricus* accessions (Table S1) were selected based on a previous screening for agronomic traits in 28 *E. sibiricus* accessions [37]. The materials seeds were germinated in the petri dish for 30 days, and then transplanted to 10 cm (diameter) flowerpots filling with 40% peat soil, 40% vermiculite, and 20% washed sand. The seedlings were grown in the greenhouse under 12 h of a photoperiod at 28 °C/14 °C day and night temperatures and 30% relative air humidity. Routine management was carried out during the whole process of growth.

### 2.2. Treatments and Tissue Collection

Six accessions were used to analyze the expression of reference genes, and three clones with consistent growth of each genotype were selected as three biological replicates. A total of 138 samples were collected from different genotypes, developmental stages, organs, and stress treatments (Table 1).

Specially, for gene expression analysis across different genotypes, leaf samples were collected from six different genotypes (ZN, XH, MQ, HZ, LQ, and LT) at 4–6 leaves stages. Leaf samples were collected from genotype ZN at different developmental stages: seedling, tillering, jointing, heading, and flowering. Tissue samples were collected from root, stem, leaf, and inflorescence at the flowering stage. For stress treatment, 4–6 leaf stage plants were removed from the soil and transferred to Hoagland’s solution. For salt treatment, plants were treated by adding 250 mmol/L NaCl to Hoagland’s solution for 0, 12, and 24 h. For osmotic stress, plants were treated by adding 20% PEG6000 to Hoagland’s solution for 0, 12, and 24 h. For heat stress, plants were moved to a growth chamber at 40 °C for 0, 12, and 24 h. For cold stress, plants were moved to another growth chamber at 3 °C for 0, 12, and 24 h. All samples were frozen in liquid nitrogen and stored in a −80 °C refrigerator for later use. 

### 2.3. RNA Extraction

Total RNA was extracted from each tissue using the plant Total RNA Kit (Sangon Biotech, Shanghai, China), according to the manufacturer’s instructions. The concentration and purity of extracted RNA were measured by NanoDrop ND-1000 spectrophotometer (NanoDrop Technologies, Wilmington, DE, USA). Only the RNA absorbing ratio of 1.8–2.0 at OD260 nm/OD280 nm and 2.0–2.6 at OD260 nm/OD230 nm were used for subsequent analysis. In addition, the integrity of RNA was evaluated by 1% agarose gel electrophoresis.

### 2.4. First-Strand cDNA Synthesis

The cDNA synthesis was performed with the M-MLV cDNA synthesis kit (Sangon Biotech, Shanghai, China), according to the manufacturer’s instructions. First, one microgram RNA was mixed with 1 μL oligonucleotide dT primer (0.5 µg/µL) in a 1.5 mL centrifuge tube, and then RNase free double distilled water was added to a final volume of 12 μL. Second, the reaction mixture was centrifuged for 3 to 5 s. The tube was incubated at 65 °C for 5 min, which was followed by cooling in ice cubes for 30 sec. Then, the mixture was centrifuged for 3–5 s. Third, the 5× Reaction Buffer 4 μL, RNase Inhibitor (40 U/μL) 1 μL, dNTP Mix (10 mmol/L) 2 μL, and M-MuLV RT (200 U/μL) 1 μL were added to the tube. Then the reaction mixture was centrifuged for 3–5 s. The first-strand cDNA was synthesized by incubating at 42 °C for 45 min. Lastly, reverse transcription was terminated by heating the reaction mixture for 10 min at 70 °C. The cDNA sample was stored at −80 °C and diluted 1:20 using RNase free water before RT-qPCR analysis.

### 2.5. Selection of Candidate Reference Genes and Primer Design

In this study, we selected 13 potential reference genes and one target gene (Table 2). These genes were obtained from our transcriptome database (https://www.ncbi.nlm.nih.gov/sra/SRX2617497) [7] by BLAST search using the sequences of reported *Arabidopsis* and rice reference genes. The 14 genes were named based on their similarity to known nucleotide sequences with the BLAST score value greater than 600 and identity ranging from 89% to 97% [33]. The primers for RT-qPCR were designed via Primer Premier 6.0 using the following parameters: melting temperature (Tm) at 58–62 °C (optimum Tm of 60 °C), PCR product length at 100–300 bp, GC content at 45–55%, and length of primers at 18–25 bp (Table 3).

### 2.6. RT-qPCR

We conducted RT-qPCR in 96-well blocks with a Bio-Rad CFX96 real-time PCR system (Bio-Rad, Hercules, CA, USA). The final reaction volume was 10 μL, and each reaction mix contained 2xSG Fast qPCR Master Mix (Low Rox) (Sangon Biotech, Shanghai, China) 5 µL, 10 μM Forward Primer 0.2 µL, 10 μM Reverse Primer 0.2 µL, 30 ng/µL cDNA 1 µL, DNF Buffer 1 µL, and ddH_2_O 2.6 µL. The amplification procedure included a denaturation step at 95 °C for 3 min, which was followed by 40 cycles of 95 °C for 3 s and 60 °C for 30 s. After the cycling process, the melting curves of RT-qPCR amplifications were obtained by raising the temperature from 60 °C to 95 °C. We also selected target gene *CSLE6* to assess the expression stability of reference genes. Each RT-qPCR reaction was carried out for the independent sample with three technical replicates.

### 2.7. Data Analysis

In the present study, the expression stability of candidate reference genes was evaluated with four algorithms: (geNorm v3.5) (https://genorm.cmgg.be/) [38], (NormFinder v0.953) (https://moma.dk/normfinder-software) [39], (BestKeeper v1.0) (https://www.gene-quantification.de/bestkeeper.html) [40], and Delta Ct [41], and then a comprehensive ranking was obtained by the RefFinder program [42]. The raw RT-qPCR data were obtained by the CFX equipment software, and the Cq (cycle quantification) values were used for further analysis. For geNorm and NormFinder methods, raw Cq values were converted into the relative quantities, according to the formula 2^−ΔCq^ (ΔCq = each corresponding Cq value—lowest Cq value) [43]. The expression stability (M) of potential reference genes was calculated by the geNorm algorithm based on the average pairwise variation of each gene with all other control genes [38]. Genes with lower M values reflect more expression stability. To determine the optimal number of reference genes for normalization, the pairwise variations (V) were calculated by geNorm. If the V_n_/V_n+1_ (*n* is the number of reference genes) value is below or equal to 0.15, the number of suitable reference genes is equal to n. The degree of variance within and between groups was evaluated via an ANOVA-based model in the NormFinder program, and the gene with the lowest stability value has the most stable expression [39]. For the BestKeeper method, the standard deviation (SD), the coefficient of variation (CV), and correlation coefficient (*r*) were calculated by Cq values and the more stable reference gene possessed the lower SD value [40]. The Delta Ct algorithm used the standard deviation (SD) to rank the stability of all reference genes, and the reference gene with the lowest SD value showed the most stable performance. The RefFinder is a web-based analysis tool. It can integrate the results from Delta Ct, BestKeeper, NormFinder, and geNorm analysis to generate a comprehensive ranking. The RefFinder program (https://github.com/fulxie/RefFinder) running in my computer works as a local server, and we deployed it to a Php-based server (Apache + PHP) at first. The standard curves were generated by using a series of two-fold diluted cDNA as templates for each primer. The correlation coefficient (*R^2^*) and slope were obtained from the linear regression model in Microsoft Excel 2016, and the PCR efficiency (E) was calculated according to the formula: E = (10^−1/slope^ − 1) × 100% [44]. The regression coefficient (R^2^) should be greater than 0.98 with the amplification efficiency (E) over 90% but less than 110%. To calculate the relative expression level of the target gene, the 2^−ΔΔCt^ method [45] was applied, and the significant difference analysis was conducted by the SPSS statistical software v20.0.

## 3. Results

### 3.1. Verification of Primer Specificity and PCR Amplification Efficiency

**Figure 1:**
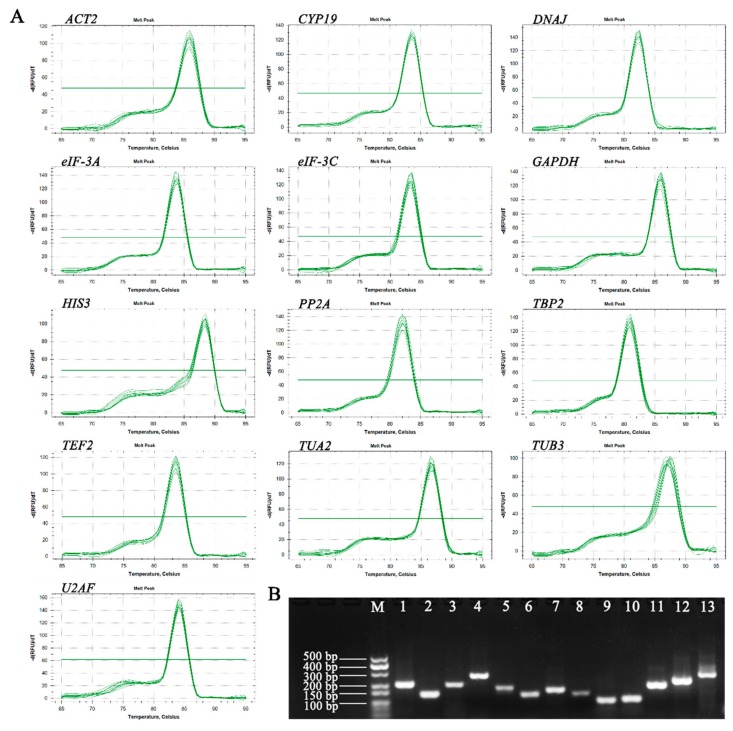
Primer specificity and amplicon size of 13 candidate reference genes. (**A**) Melting curves of 13 candidate reference genes exhibiting single peaks. (**B**) Agarose gel electrophoresis (2%) showing specific amplification products of expected size using Real Time-qPCR. M: 500 bp marker. 1–13: *ACT2*, *CYP19*, *DNAJ*, *eIF-3A*, *eIF-3C*, *GAPDH*, *HIS3*, *PP2A*, *TBP2*, *TEF2*, *TUA2*, *TUB3*, and *U2AF*.

In the present study, we selected 13 potential reference genes based on transcriptome data of *E. sibiricus* to investigate the expression stability under different conditions. Subsequently, specific primers for RT-qPCR were designed according to the sequences of 13 candidate reference genes. In order to check the specificity of all primers, agarose gel electrophoresis (2%) and melting curve analysis of the amplification products were conducted (Figure 1). The results of agarose gel electrophoresis showed that each primer amplified a single product of the expected size. Additionally, a single peak for each primer was observed from the melting curve analysis. These results indicated that all of the primers in this experiment were specific. The amplification efficiency (E) of 14 primers varied from 90.35% (*DNAJ*) to 101.08% (*PP2A*), and the regression coefficient (R^2^) ranged from 0.981 (*ACT2*) to 0.998 (*GAPDH*, *TUB3* and *U2AF*).

### 3.2. Cq Data Collection and Variations in Reference Genes

**Figure 2:**
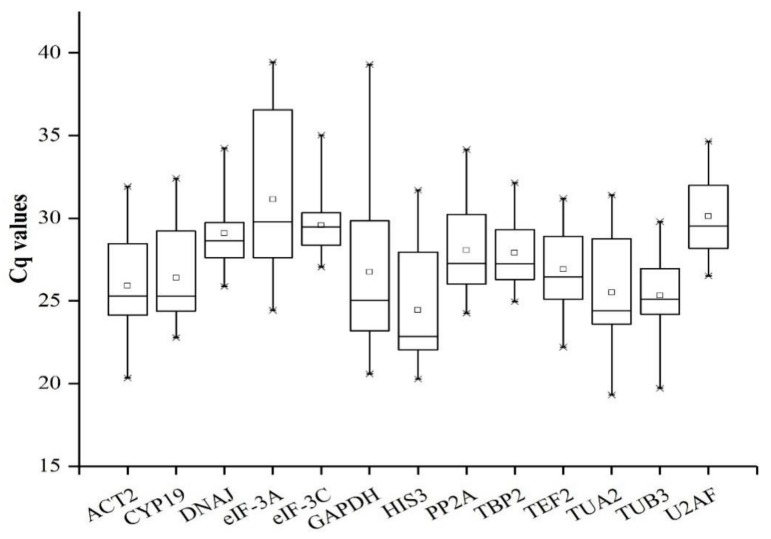
RT-qPCR Cq values for all candidate reference genes in all *E. sibiricus* samples. Whisker caps, boxes, lines, and square boxes represent maximum/minimum, 25/75 percentiles, median, and mean, respectively.

In order to analyze the expression level of all potential reference genes under different experimental conditions, the Cq values of all samples were obtained by RT-qPCR (Figure 2). The Cq values of 13 candidate reference genes ranged from 19.3 (*TUA2* expressed in the stem sample) to 39.44 (*eIF-3A* expressed in the root sample under osmotic stress for 24 h) among all samples. Furthermore, the top three highly expressed genes were *HIS3* (mean Cq = 24.45), *TUB3* (mean Cq = 25.32), and *TUA2* (mean Cq = 25.49). Three lowly expressed genes were *eIF-3A* (mean Cq = 31.14), *U2AF* (mean Cq = 30.12), and *eIF-3C* (mean Cq = 29.57). The coefficient of variation (CV) of the Cq values reflects the expression stability of the reference gene, and the reference gene with a low CV value represents a high degree of stable expression. Therefore, *eIF-3C* (CV = 5.40%) possessed the most stable expression due to the lowest CV value, and the *GAPDH* (CV = 18.87%) with the highest CV value had the most unstable expression.

### 3.3. Expression Stability Analysis of the Candidate Reference Genes

To determine the most stable reference gene of *E. sibiricus*, four common algorithms (geNorm, NormFinder, BestKeeper, and Delta Ct) [38,39,40,41] and an integrated analysis tool (RefFinder) [42] were applied to assess the expression stability of all candidate reference genes. Moreover, the top six stable reference genes from the four algorithms were shown in Figure S1.

#### 3.3.1. GeNorm Analysis

**Figure 3:**
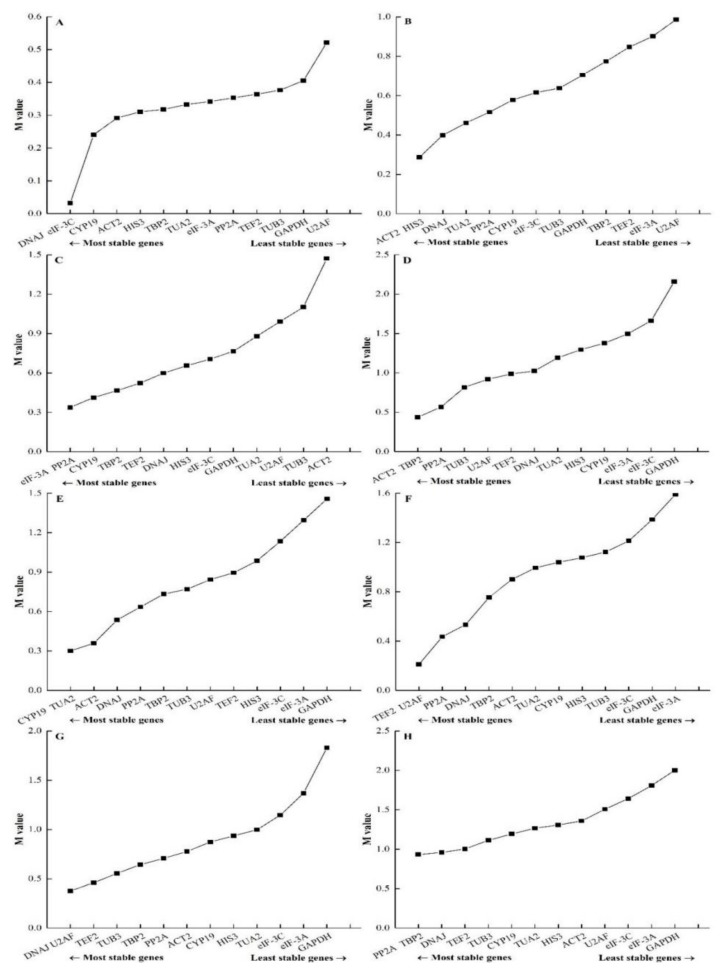
Average expression stability values (M) of 13 candidate reference genes under different conditions calculated by geNorm. (**A**) Different genotypes, (**B**) different developmental stages, (**C**) different tissues, (**D**) salt stress, (**E**) heat stress, (**F**) cold stress, (**G**) osmotic stress, and (**H**) all samples.

The expression stability of 13 potential reference genes in the different genotypes, developmental stages, tissues, and different abiotic stress treatments was evaluated by the geNorm algorithm (Figure 3). According to the threshold of M value recommended by the geNorm program, a candidate gene could be used as a reference gene for expression analysis only when its M value is under 1.5. Our results showed that *DNAJ* and *eIF-3C* were the most stable genes with the lowest M value (0.03) in different genotypes, while *U2AF* was the least stable gene with the highest M value (0.52) (Figure 3A). In different developmental stage samples (Figure 3B), the most stable genes were *ACT2* and *HIS3* (M = 0.29), while *U2AF* was the least stable gene (M = 0.99). *eIF-3A* and *PP2A* (M = 0.34) showed the most stability while *ACT2* (M = 1.47) was identified as the least stable gene in different tissues (Figure 3C). *ACT2* and *TBP2* (M = 0.44) were the most stable genes under salt stress (Figure 3D), while *GAPDH* (M = 2.16) was the least stable gene. *CYP19* and *TUA2* (M = 0.30) had the most stability, whereas *GAPDH* (M = 1.46) had the worst stability in heat stress samples (Figure 3E). For the cold stress group (Figure 3F), *TEF2* and *U2AF* (M = 0.21) were the most stable genes while *eIF-3A* (M = 1.59) was the least stable gene. Under osmotic stress (Figure 3G), *DNAJ* and *U2AF* (M = 0.38) showed the highest degree of expression stability while *GAPDH* (M = 1.83) presented the lowest degree of expression stability. Meanwhile, *PP2A* and *TBP2* (M = 0.93) were the most stable genes in all samples and *GAPDH* (M = 2.00) was the least stable gene. In summary, the most stable gene was different in various experimental sample sets.

**Figure 4:**
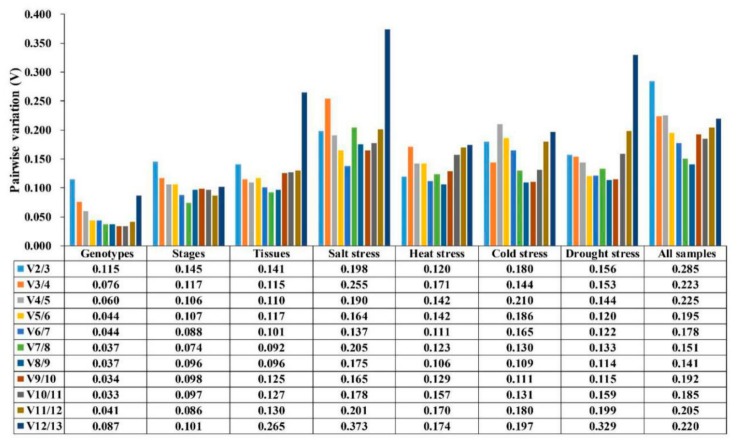
Pairwise variation (V) of 13 candidate reference genes under various experimental conditions calculated by geNorm.

In order to obtain the optimal number of reference genes in different conditions, pairwise variation (V) was calculated by the geNorm program (Figure 4). Moreover, 0.15 was used as the threshold value to determine the optimal number of reference genes. For non-stress treatment groups (different genotypes, different developmental stages and different tissues), all pairwise variations except for V_12/13_ (0.265) of different tissues were lower than 0.15, which demonstrated that one reference gene was sufficient for gene expression analysis. For the stress groups (salt stress, heat stress, cold stress, and osmotic stress), however, it showed different results. The V_6/7_ (0.137) value from salt stress was lower than 0.15, which indicated that the top six reference genes (*ACT2*, *TBP2*, *PP2A*, *TUB3*, *U2AF,* and *TEF2*) were required for expression analysis. The V_2/3_ (0.120) value from heat stress samples was lower than 0.15, which suggested that the top two reference genes (*CYP19* and *TUA2*) were sufficient for normalization. The V_3/4_ (0.144) value from the cold stress group was lower than 0.15, which indicated that the top three genes (*TEF2*, *U2AF*, and *PP2A*) were required for normalization. Nevertheless, the threshold value of 0.15 should not be regarded as a rigorous standard, and higher cut-off values of V_n_/V_n+1_ were also found in several reports [35,43,46]. A minor variation was found between V_2/3_ (0.156) and V_3/4_ (0.153) in samples from osmotic stress, which revealed that two reference genes (*DNAJ* and *U2AF*) were sufficient for expression analysis. Similarly, V_3/4_ (0.223) and V_4/5_ (0.225) from all samples showed slight variation, which illustrates that the top three genes (*PP2A*, *TBP2*, and *DNAJ*) were needed for normalization.

#### 3.3.2. NormFinder Analysis

**Figure 5:**
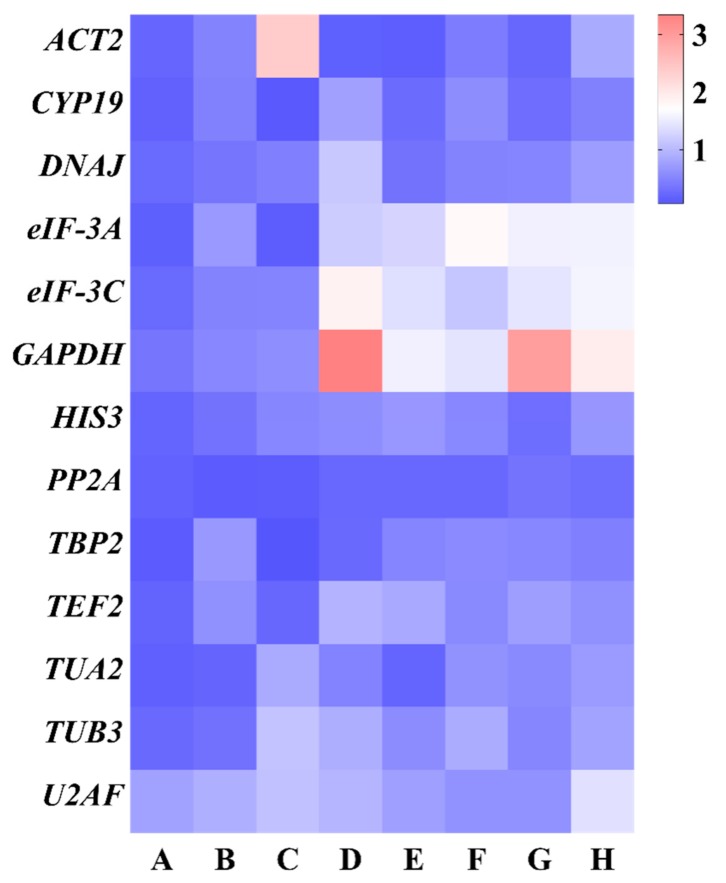
Expression stability of *E. sibiricus* potential reference genes calculated using NormFinder. (**A**) Different genotypes, (**B**) different developmental stages, (**C**) different tissues, (**D**) salt stress, (**E**) heat stress, (**F**) cold stress, (**G**) osmotic stress, and (**H**) all samples. Low to high expression stability is represented over a spectrum from red to blue, respectively.

The 13 candidate reference genes were ranked based on the expression stability value calculated using the NormFinder program (Figure 5 and Table S2). The most stable gene possessed the highest expression stability value. Accordingly, *TBP2* had the highest ranking in different genotypes and different tissues with the stability values of 0.10 and 0.07, respectively. *PP2A* was the most stable reference gene in different developmental stages, cold stress, and all samples with the stability values of 0.11, 0.23 and 0.29, respectively. As for the salt stress, heat stress, and osmotic stress, *ACT2* was identified as the most stably expressed reference genes with the stability values of 0.15, 0.13, and 0.22, respectively. Although the most stable reference genes according to the NormFinder algorithm were different from that of geNorm in most of the experimental sets, the least stable reference genes in all groups were consistent between NormFinder and geNorm. For example, according to the geNorm analysis, *DNAJ* and *CYP19* were the most stable reference genes in different genotypes and heat stress, whereas their stability rankings were ninth and fourth in the NormFinder analysis, respectively. Furthermore, *U2AF, ACT2, GAPDH*, and *eIF-3A* had lower expression stability among all experimental groups by using the geNorm and NormFinder program.

#### 3.3.3. BestKeeper Analysis

**Figure 6:**
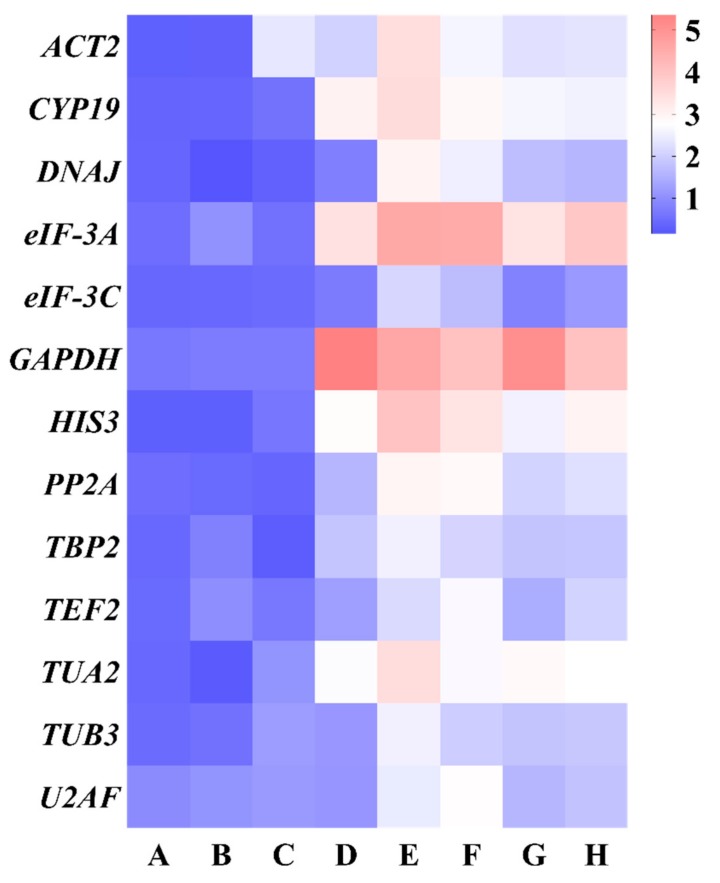
Expression stability of candidate reference genes calculated by BestKeeper. (**A**) Different genotypes, (**B**) different developmental stages, (**C**) different tissues, (**D**) salt stress, (**E**) heat stress, (**F**) cold stress, (**G**) osmotic stress, and (**H**) all samples. Low to high expression stability is represented over a spectrum from red to blue, respectively.

The BestKeeper program was used to analyze the expression stability of 13 reference genes according to the values of standard deviation (SD), the coefficient of variation (CV), and correlation coefficient (r). The lower SD value of genes represented the higher expression stability (Figure 6 and Table S3). Hence, *HIS3* was the most stable reference gene in different genotypes, while it ranked fifth and seventh in geNorm and NormFinder analysis, respectively. *DNAJ* exhibited the best expression stability in different developmental stages, which ranked third and fifth in geNorm and NormFinder analysis, respectively. In different tissues, *TBP2* was the most stable reference gene in both BestKeeper and NormFinder analysis, but it ranked fourth in geNorm analysis. For salt stress, heat stress, cold stress, osmotic stress, and all samples, *eIF-3C* presented the most stable expression with the lowest SD values, while it had low ranking in geNorm and NormFinder analysis. *U2AF*, *ACT2*, *GAPDH*, and *eIF-3A* were considered to be the least stable genes in all experimental conditions due to their highest SD values, which was consistent with the results from geNorm and NormFinder analysis.

#### 3.3.4. Delta Ct Analysis

**Figure 7:**
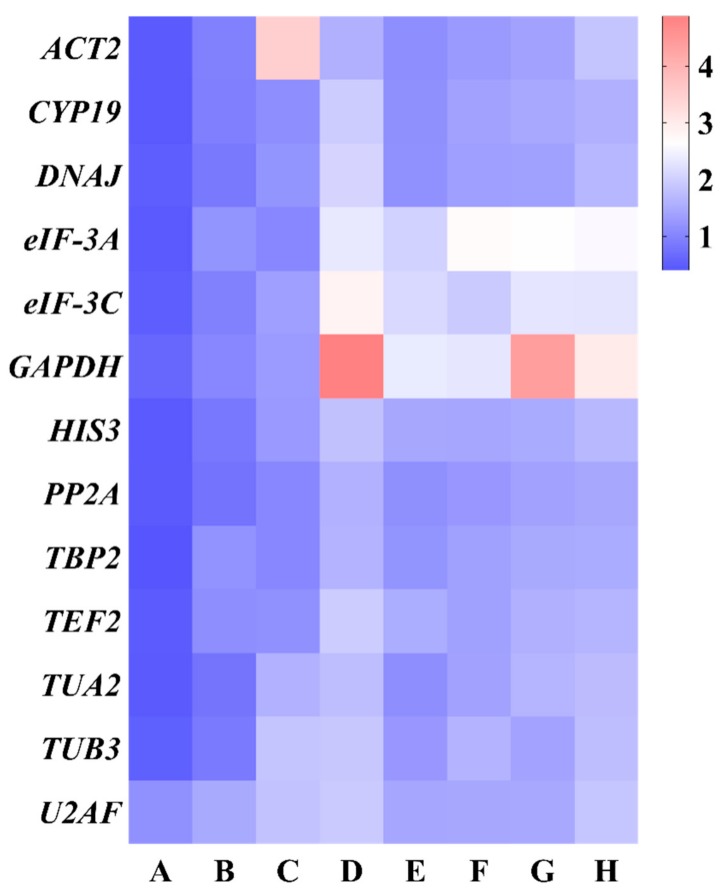
Expression stability for 13 candidate reference genes calculated via Delta Ct. (**A**) Different genotypes, (**B**) different developmental stages, (**C**) different tissues, (**D**) salt stress, (**E**) heat stress, (**F**) cold stress, (**G**) osmotic stress, and (**H**) all samples. Low to high expression stability is represented over a spectrum from red to blue, respectively.

The values of standard deviation (SD) was used as the indicator for evaluating the expression stability of reference genes using the Delta Ct method. A gene with the lowest SD value indicated the most stable reference gene (Figure 7 and Table S4). Accordingly, *TBP2*, *PP2A*, *ACT2*, and *DNAJ* showed the most stable expression in experimental conditions. Meanwhile, *U2AF*, *ACT2*, *GAPDH*, and *eIF-3A* were the least stable genes in all sample sets, which was consistent with geNorm, NormFinder, and BestKeeper analysis.

#### 3.3.5. RefFinder Analysis

RefFinder, which is a comprehensive analysis tool for expression stability of reference genes, was used to calculate the synthetic rankings of 13 potential reference genes based on four approaches including geNorm, NormFinder, BestKeeper, and Delta Ct (Figure S2 and Table 4). For different genotypes, *TBP2*, *HIS3*, and *CYP19* were the top three stable genes according to the RefFinder algorithm analysis. *TUA6, PP2A*, and *HIS3* were considered as the three most stable genes for different developmental stages. In different tissues, *TBP2*, *PP2A*, and *eIF-3A* showed high expression stability. *ACT2*, *TBP2*, and *PP2A* were identified as the most stably expressed reference genes under salt stress. *ACT2*, *TUA2*, and *CYP19* had the best expression stability under heat stress. *PP2A*, *ACT2*, and *DNAJ* exhibited a high level of expression stability under cold stress. As for osmotic stress, *DNAJ*, *U2AF*, and *ACT2* were considered as the most stable genes. For all samples, *PP2A*, *TBP2*, and *DNAJ* were the most stable genes. However, *U2AF* was the least stable reference genes in different genotypes and different developmental stages. *ACT2* was identified as the least stable reference genes in different tissues. Under salt stress, heat stress, and osmotic stress and for all samples, *GAPDH* had the worst expression stability. *eIF-3A* showed the lowest level of expression stability under cold stress.

The results of the above five methods indicated that the most stable genes differed when different experimental sample sets were compared, which illustrates the significance of suitable reference genes that are specific to each experimental condition. Therefore, the top two stable reference genes and the least reference gene in different experimental conditions were selected for validating the expression stability of reference genes, according to the RefFinder analysis.

### 3.4. Validation of the Stability of Reference Genes

**Figure 8:**
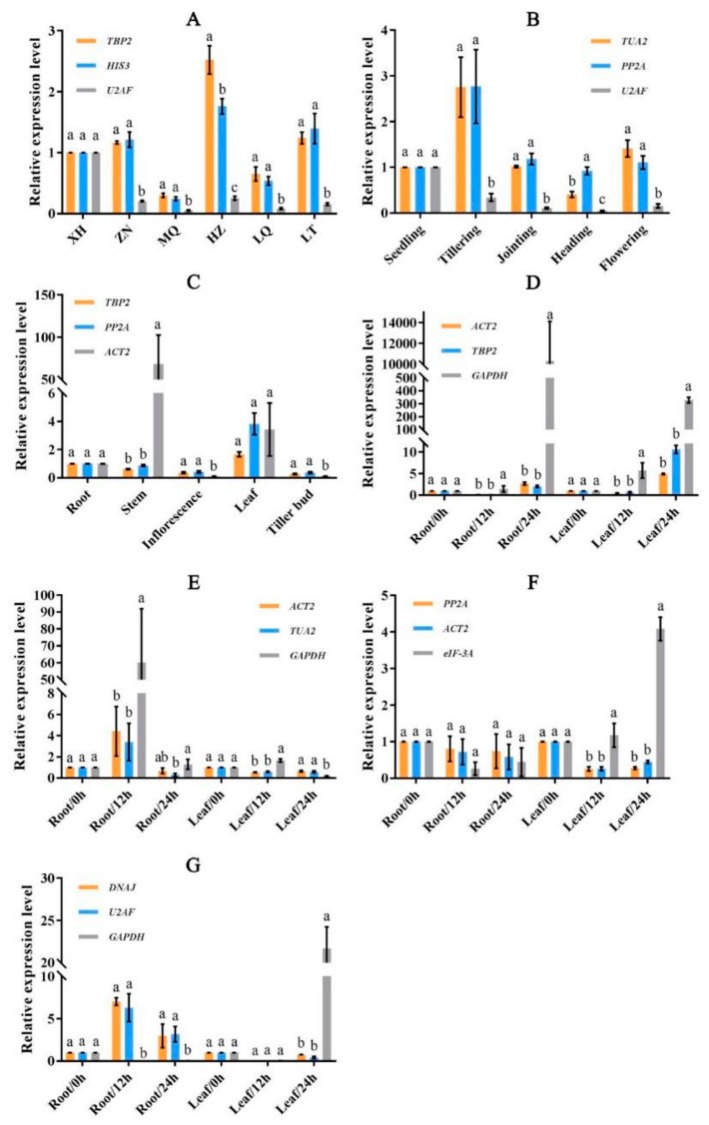
The relative expression level of target gene *CSLE6* in various experimental conditions. The most two stable reference genes and the most unstable reference genes in different conditions were selected for expression normalization. (**A**) Different genotypes, (**B**) different developmental stages, (**C**) different tissues, (**D**) salt stress, (**E**) heat stress, (**F**) cold stress, and (**G**) osmotic stress. Different letters in the same sample represent a significant difference among three reference genes at the 0.05 level. Error bars indicate standard deviation.

In order to detect the performance of expression stability among reference genes, we selected two reference genes with a high degree of stability and one unstable gene to analyze the expression patterns of Cellulose synthase-like protein E6 gene (*CSLE6*) under different experimental conditions (Figure 8). The reference genes were selected for expression normalization in different experimental conditions as follows: *TBP2*, *HIS3*, and *U2AF* for different genotypes, *TUA2*, *PP2A*, and *U2AF* for different developmental stages, *TBP2*, *PP2A*, and *ACT2* for different tissues, *ACT2*, *TBP2*, and *GAPDH* for salt stress, *ACT2*, *TUA2*, and *GAPDH* for heat stress, *PP2A*, *ACT2*, and *eIF-3A* for cold stress, *DNAJ*, *U2AF*, and *GAPDH* for osmotic stress. Meanwhile, the 2^−ΔΔCt^ method was used to calculate the relative expression level of target gene *CSLE6*.

As shown in Figure 8, the expression patterns of *CSLE6* generated by two stable reference genes were similar. In contrast, the relative expression level of *CSLE6* showed abnormal trends when the unstable reference genes were selected for expression normalization. Moreover, there was no significant difference (*p* < 0.05) in the relative expression level of *CSLE6* between two stable reference genes in most of the sample sets, while the expression trend of *CSLE6* derived from unstable reference genes was significantly different from that of stable reference genes. For example, the relative expression level of *CSLE6* in HZ (Figure 8A) and tillering (Figure 8B) samples were underestimated, and abnormally up-regulated expressions were found in the stem sample (Figure 8C), the root and leaf samples under salt stress at 24 h (Figure 8D), the root sample under heat stress at 12 h (Figure 8E), the leaf sample under cold stress (Figure 8F), and osmotic stress (Figure 8G) at 24 h. Furthermore, the expression pattern analysis of *CSLE6* exhibited that *CSLE6* had a high expression level in the HZ genotype sample, the tillering sample, and the leaf sample with non-stress treatment. The target gene also had high expression in the leaf sample under salt stress at 24 h, and in the root sample under heat and osmotic stress at 12 h. The expression level of *CSLE6* was down-regulated in leaf and root samples under cold stress.

## 4. Discussion

RT-qPCR is an effective technique for gene expression analysis due to its high accuracy, simplicity, specificity, and sensitivity [8,9,10]. It is reported that inappropriate reference genes will lead to opposite conclusions in gene expression analysis [24]. Consequently, evaluating the expression stability of potential reference genes is necessary to quantify the expression level of target genes, and to analyze the expression pattern of genes of interest [47]. Numerous studies suggested that none of the reference genes maintain the consistent expression stability among various experimental conditions, and it is imperative to carry out reference gene screening under specific experimental conditions [11,29,48,49,50,51]. To our knowledge, there have been no studies regarding the selection of appropriate reference genes in *E. sibiricus*, and, thus, this study is the first report with respect to the systematic selection and evaluation of reference genes for RT-qPCR normalization in *E. sibiricus*.

In the present study, 13 candidate reference genes were selected from transcriptome sequencing data to evaluate their expression stability in different experimental conditions [7]. The results showed that all reference gene primers possessed good amplification efficiency (90.35% to 101.08%), regression coefficient (0.981 to 0.998) and Cq values (25 to 30), which illustrates that the RT-qPCR data are suitable for further analysis. Four commonly used methods including geNorm, NormFinder, BestKeeper, and Delta Ct [38,39,40,41] were used to assess the expression stability of 13 candidate reference genes. The results suggested that the least stable reference genes of the same experimental samples were consistent among four algorithms (Table 4), while the most stable reference genes were inconsistent in different conditions (Figure S1 and Table 4). For example, *U2AF* and *ACT2* were unstable reference genes based on four algorithms in non-stress conditions. *GAPDH* and *eIF-3A* also had the worst stable performance under stress conditions. Furthermore, *DNAJ* was the most stable reference gene found by the geNorm algorithm in different genotypes, while it ranked ninth in NormFinder and Delta Ct analysis. The *eIF-3C* had the highest expression stability by the BestKeeper algorithm in four stress conditions, but it was considered the unstable reference gene in geNorm, NormFinder, and Delta Ct analysis. The heterogeneous result was also found in previous studies, and that may be caused by different algorithms [29,52]. Fortunately, we obtained an integrated evaluation result of potential reference genes by using RefFinder. RefFinder is regarded as a comprehensive analysis tool, which has extensive recognition in determining the optimal reference genes for gene expression analysis [12,21,53,54,55]. Lastly, we adopted the rankings derived from the RefFinder method as the ultimate evaluation results of expression stability of candidate reference genes, and selected suitable reference genes for validating the expression stability based on the results of RefFinder.

Previous studies demonstrated that there are no any reference genes with consistent expression stability among different conditions [11,29,48,49,50,51]. Our results were similar to previous studies. For example, *ACT2* exhibited a high degree of expression stability under four stress conditions, whereas *ACT2* was the least stable reference gene in different tissues. *TUA2* was the most stable reference gene in different developmental stages, but it ranked eleventh under osmotic stress. These results indicated that the importance of selecting appropriate reference genes in different experimental conditions.

The plant cell wall provides a guarantee for plants to adapt to various environmental conditions. Cellulose, which is the main component of cell walls in the plant, is synthesized by cellulose synthases (*CesA*) [56]. A considerable number of cellulose synthase-like (*CSL*) genes have similarities with *CesA* gene [57], and, thus, the *CSL* gene also plays an important role in cellulose synthesis [58]. Previous research studies indicated that the expressions of cellulose synthesis genes are closely related to the adaptation of plants under different stress conditions [56]. In addition, other studies showed that cellulose synthesis genes were associated with shedding or shattering in leaves, flowers, fruits, and seed [7,59,60]. *CSLE6*, which is a member of the superfamily of genes referred to as glycosyltransferase 2 (GT2), has the function of synthesizing cellulose in the plant cell wall [61]. However, there are few studies regarding the *CSLE6* gene. This gene was identified in *Arabidopsis* (At1g55850, named *AtCSLE1*) and rice (LOC_Os09g30130, named *OsCSLE6*), respectively [57,62]. In the present study, to validate the expression stability of reference genes, we selected *CSLE6* as a target gene to investigate the expression pattern of *CSLE6* in different conditions. Several previous reports suggested that selecting two or more stable reference genes to calculate the relative expression levels of target genes will generate more reliable results [21,25,26,63]. Hence, we selected two stable reference genes and the least stable reference gene in different conditions for the expression pattern analysis of *CSLE6*. As shown in Figure 8, *CSLE6* exhibited distinct expression levels in different experimental conditions. In different genotypes, the expression level of *CSLE6* in the ZN (low seed shattering) genotype was higher than the XH genotype (high seed shattering). This is similar to the *OsCel9D* [60], which is a seed shattering related gene. This gene indicates that *CSLE6* may have similar functions in seed shattering. On the one hand, *CSLE6* directly hampers the abscission process in seed shattering by changing the cell wall components, such as cellulose content and pectin content [60]. On the other hand, *CSLE6* indirectly reduces the seed shattering by affecting the lignin biosynthesis that is closely related to seed shattering [64,65]. In different developmental stages, the order of expression levels of *CSLE6* was tillering > flowering > jointing > seedling > heading. This is different from the previous study of *E. sibiricus* [7], likely due to different tissues and periods. Plants generate a large number of new leaves and cells during the tillering stage, which may explain that *CSLE6* had the highest expression in the tillering stage. For different tissues, the order of expression levels of *CSLE6* is leaf > root > stem > inflorescence > tiller bud. This was similar to *Arabidopsis* [62], which the highest expression was found in the leaf sample. This was followed by the root sample. The leaf samples in this analysis were not young leaves (flowering stage) and the cellulose accounts for a high proportion in the cell wall, where the high expression of *CSLE6* may play a role in replacing the homogalacturonan (HGA) in the cell wall [62,66]. Furthermore, previous reports revealed that cellulose synthesis genes usually have high expression in tissues with cell division and expansion, such as root and hypocotyl [64,67]. Under salt stress, the expression of *CSLE6* in leaf and root samples showed a trend of decreasing first and then increasing, which is different from that in *Arabidopsis* [68]. For cold stress, the expression level of *CSLE6* was down-regulated in leaf and root samples. Many studies indicated that abiotic stress (e.g., salt stress and cold stress) will reduce the cell expansion and inhibit plant growth [69,70,71]. In addition, the expression levels of a great number of cellulose synthesis genes were reduced by cold stress or salt stress in *Arabidopsis* [62,67], poplar [72], and cotton [73], while these genes showed increased expression in rice [74]. Hence, the cellulose synthesis genes have distinct expression patterns in different experimental conditions and different species because of the multiple ways in which plants adapt to the environment. More importantly, the above results illustrated that the diverse expression patterns of *CSLE6* reflected the adaptation mechanisms for *E. sibiricus* under a variety of environmental conditions.

## 5. Conclusions

In summary, the most stable reference genes were different under distinct experimental conditions in this study. To obtain the precise results of gene expression analysis, it is recommended to adopt suitable reference genes in specific experimental conditions. One or more stable reference genes should be selected to investigate the expression pattern of target genes based on the comprehensive evaluation results from RefFinder. The expression pattern of *CSLE6* may provide a basis for studying the resistance mechanism in *E*. *sibiricus*. Moreover, our study screened several suitable reference genes in specific conditions for *E*. *sibiricus*, and offered some guidelines for the selection of reference genes for other plant species.

## Figures and Tables

**Table 1:** Experimental sets in the present study.

Sample Sets	Tissue Type	Sampling Dates	Germplasm	Biological Replicates	Total Number of Samples
Different genotypes	Leaf	4–6 leaves stage	ZN, XH, MQ, HZ, LQ, LT	3	18
Different developmental stages	Leaf	Seedling, Tillering, Jointing, Heading, Flowering	ZN	3	15
Different tissues	Root, Stem, Leaf, Tiller bud, Inflorescence	Flowering stage	ZN	3	15
Salt stress (250 mmol/L NaCl)	Leaf, Root	4–6 leaves stage, 0, 12, 24 HAT ^1^	ZN	3	18
Cold stress (3 °C)	Leaf, Root	4–6 leaves stage, 0, 12, 24 HAT	ZN	3	18
Heat stress (40 °C)	Leaf, Root	4–6 leaves stage, 0, 12, 24 HAT	ZN	3	18
Osmotic stress (20% PEG6000)	Leaf, Root	4–6 leaves stage, 0, 12, 24 HAT	ZN	3	18
Control	Leaf, Root	4–6 leaves stage, 0, 12, 24 HAT	ZN	3	18

Abbreviation:^1^ HAT, hours after treatment.

**Table 2:** Description of 13 reference genes and one target gene.

Gene	Gene Description	*Arabidopsis* Homolog Locus	Amino Acid Identity with *E. sibiricus* (%)	Rice TIRG Identifier	Amino Acid Identity with *E. sibiricus* (%)
*ACT2*	Actin2	At5g09810	98.41	LOC_Os11g06390	98.94
*CYP19*	Cyclophilin 19	At2g29960	75.12	LOC_Os06g49480	82.73
*DNAJ*	*DNAJ* heat shock N-terminal domain-containing protein	At1g76700	66.67	LOC_Os08g41110	86.04
*eIF-3A*	Eukaryotic translation initiation factor 3A	At4g11420	62.36	LOC_Os01g03070	79.39
*eIF-3C*	Eukaryotic translation initiation factor 3C	At3g56150	62.18	LOC_Os07g03230	82.43
*GAPDH*	Glyceraldehyde-3-phosphate dehydrogenase	At3g04120	74.93	LOC_Os08g03290	92.12
*HIS3*	Histone 3.3	At4g40030	82.93	LOC_Os06g04030	100.00
*PP2A*	Protein phosphatase 2A	At1g13320	74.28	LOC_Os09g07510	78.36
*TBP2*	TATA binding protein 2	At1g55520	64.50	LOC_Os03g45410	59.65
*TEF2*	Translation elongation factor 2	At1g56070	91.10	LOC_Os02g32030	94.90
*TUA2*	Tubulin α-2	At4g14960	96.67	LOC_Os11g14220	97.56
*TUB3*	Tubulin β-3	At1g75780	89.04	LOC_Os06g46000	90.13
*U2AF*	U2 auxiliary factor	At5g42820	74.48	LOC_Os09g31482	69.97
***CSLE6***	Cellulose synthase-like protein E6	At1g55850	47.92	LOC_Os09g30130	76.64

**Table 3:** Details of primers and amplicons for 14 genes.

Candidate Reference Gene	Primer Sequence F/R (5′–3′)	Tm (°C)	Amplicon Length/Bp	Efficiency (%)	R^2^
*ACT2*	F: CCACGAGACGACCTACAATTCCATC	60.0	206	98.46	0.981
	R: CTCCGATCCAGACACTGTACTTCCT				
*CYP19*	F: GGTGGTGAATCAATCTACGGCACAA	60.4	141	93.73	0.996
	R: GCTCGTGGTTACAGTGGTGATGAAG				
*DNAJ*	F: GCAATGGCGTCAATGGCTTCAC	59.6	209	90.35	0.992
	R: GCATCACTAAGTCTGGACACCTCAG				
*eIF-3A*	F: GAATCAGGCACAAGCACTGGAAGA	59.9	284	95.4	0.984
	R: ACAACCTACGGAACTCGGTGGAT				
*eIF-3C*	F: GAATCATAAGGCTGCTGCGAAGGT	60.0	183	92.84	0.990
	R: AACGGTGGTGGTCCTCTATTGTCA				
*GAPDH*	F: GTTACTGTCTTCGGCGTCAGGAAC	60.2	137	97.05	0.998
	R: ACCTTCTTGGCACCACCCTTCA				
*HIS3*	F: CTACAACTGGAGGAGTGAAGAAGCC	59.6	164	98.85	0.993
	R: GAAGCGGAGGTCAGTCTTGAAGTC				
*PP2A*	F: GTGATAATGAGGCTGAAGTGCGGAT	60.0	140	101.08	0.990
	R: GCGAACATGCTGAGACGAATCTGA				
*TBP2*	F: CGGATGAGGCAGCCAAAGATTGT	59.6	103	98.65	0.993
	R: TGTTCTCGAAGGCAGTGTATGTCTC				
*TEF2*	F: AGAAGTCCTGCCGTACCGTTATGA	60.1	107	98.97	0.995
	R: GCCATCATCAATAGCCTCAGCCAAT				
*TUA2*	F: TGGTGATGTTGTGCCGAAGGATG	59.8	195	100.6	0.996
	R: ACGACACTGGTGGAGTTGGAGAT				
*TUB3*	F: GTGCAGAACAAGAACTCGTCCTACT	59.5	233	99.97	0.998
	R: TCGGTGAACTCCATCTCGTCCAT				
*U2AF*	F: ATCGCTGCTCTCGCATCCATAAC	59.7	281	98.5	0.998
	R: TGCTGCTGCCTGATCTTCCTCT				
Target gene					
***CSLE6***	F: AAGGATGGTGGAATGGACAGAGGAT	60.0	149	98.37	0.994
	R: CTTGGACTCGTCTTCGTCGCTTAC				

**Table 4:** Stability ranking of 13 candidate reference genes calculated by Delta Ct, BestKeeper, NormFinder, GeNorm, and RefFinder.

Method	Ranking Order (Better—Good—Average)												
	1	2	3	4	5	6	7	8	9	10	11	12	13
Different genotypes													
Delta Ct	*TBP2*	*HIS3*	*eIF-3A*	*CYP19*	*PP2A*	*ACT2*	*TUA2*	*TEF2*	*DNAJ*	*eIF-3C*	*TUB3*	*GAPDH*	*U2AF*
BestKeeper	*HIS3*	*ACT2*	*CYP19*	*DNAJ*	*eIF-3C*	*TUA2*	*TBP2*	*TEF2*	*TUB3*	*eIF-3A*	*PP2A*	*GAPDH*	*U2AF*
NormFinder	*TBP2*	*eIF-3A*	*TUA2*	*PP2A*	*CYP19*	*TEF2*	*HIS3*	*ACT2*	*DNAJ*	*eIF-3C*	*TUB3*	*GAPDH*	*U2AF*
GeNorm	*DNAJ* | *eIF-3C*		*CYP19*	*ACT2*	*HIS3*	*TBP2*	*TUA2*	*eIF-3A*	*PP2A*	*TEF2*	*TUB3*	*GAPDH*	*U2AF*
RefFinder	*TBP2*	*HIS3*	*CYP19*	*DNAJ*	*ACT2*	*eIF-3A*	*eIF-3C*	*TUA2*	*PP2A*	*TEF2*	*TUB3*	*GAPDH*	*U2AF*
Different developmental stages													
Delta Ct	*PP2A*	*TUA2*	*HIS3*	*DNAJ*	*TUB3*	*CYP19*	*eIF-3C*	*ACT2*	*GAPDH*	*TEF2*	*TBP2*	*eIF-3A*	*U2AF*
BestKeeper	*DNAJ*	*TUA2*	*HIS3*	*ACT2*	*CYP19*	*eIF-3C*	*PP2A*	*TUB3*	*GAPDH*	*TBP2*	*TEF2*	*eIF-3A*	*U2AF*
NormFinder	*PP2A*	*TUA2*	*TUB3*	*HIS3*	*DNAJ*	*CYP19*	*eIF-3C*	*ACT2*	*GAPDH*	*TEF2*	*TBP2*	*eIF-3A*	*U2AF*
GeNorm	*ACT2* | *HIS3*		*DNAJ*	*TUA2*	*PP2A*	*CYP19*	*eIF-3C*	*TUB3*	*GAPDH*	*TBP2*	*TEF2*	*eIF-3A*	*U2AF*
RefFinder	*TUA2*	*PP2A*	*HIS3*	*DNAJ*	*ACT2*	*TUB3*	*CYP19*	*eIF-3C*	*GAPDH*	*TBP2*	*TEF2*	*eIF-3A*	*U2AF*
Different tissues													
Delta Ct	*PP2A*	*TBP2*	*eIF-3A*	*CYP19*	*TEF2*	*DNAJ*	*HIS3*	*GAPDH*	*eIF-3C*	*TUA2*	*U2AF*	*TUB3*	*ACT2*
BestKeeper	*TBP2*	*DNAJ*	*PP2A*	*eIF-3C*	*eIF-3A*	*CYP19*	*HIS3*	*TEF2*	*GAPDH*	*TUA2*	*U2AF*	*TUB3*	*ACT2*
NormFinder	*TBP2*	*CYP19*	*PP2A*	*eIF-3A*	*TEF2*	*DNAJ*	*eIF-3C*	*HIS3*	*GAPDH*	*TUA2*	*U2AF*	*TUB3*	*ACT2*
GeNorm	*eIF-3A* | *PP2A*		*CYP19*	*TBP2*	*TEF2*	*DNAJ*	*HIS3*	*eIF-3C*	*GAPDH*	*TUA2*	*U2AF*	*TUB3*	*ACT2*
RefFinder	*TBP2*	*PP2A*	*eIF-3A*	*CYP19*	*DNAJ*	*TEF2*	*eIF-3C*	*HIS3*	*GAPDH*	*TUA2*	*U2AF*	*TUB3*	*ACT2*
Salt stress													
Delta Ct	*ACT2*	*PP2A*	*TBP2*	*TUA2*	*HIS3*	*TUB3*	*U2AF*	*CYP19*	*TEF2*	*DNAJ*	*eIF-3A*	*eIF-3C*	*GAPDH*
BestKeeper	*eIF-3C*	*DNAJ*	*U2AF*	*TUB3*	*TEF2*	*PP2A*	*TBP2*	*ACT2*	*TUA2*	*HIS3*	*CYP19*	*eIF-3A*	*GAPDH*
NormFinder	*ACT2*	*PP2A*	*TBP2*	*TUA2*	*HIS3*	*CYP19*	*TUB3*	*TEF2*	*U2AF*	*DNAJ*	*eIF-3A*	*eIF-3C*	*GAPDH*
GeNorm	*ACT2* | *TBP2*		*PP2A*	*TUB3*	*U2AF*	*TEF2*	*DNAJ*	*TUA2*	*HIS3*	*CYP19*	*eIF-3A*	*eIF-3C*	*GAPDH*
RefFinder	*ACT2*	*TBP2*	*PP2A*	*TUB3*	*U2AF*	*TUA2*	*DNAJ*	*eIF-3C*	*TEF2*	*HIS3*	*CYP19*	*eIF-3A*	*GAPDH*
Heat stress													
Delta Ct	*ACT2*	*TUA2*	*PP2A*	*CYP19*	*DNAJ*	*TBP2*	*TUB3*	*U2AF*	*HIS3*	*TEF2*	*eIF-3A*	*eIF-3C*	*GAPDH*
BestKeeper	*eIF-3C*	*TEF2*	*U2AF*	*TUB3*	*TBP2*	*PP2A*	*DNAJ*	*ACT2*	*TUA2*	*CYP19*	*HIS3*	*eIF-3A*	*GAPDH*
NormFinder	*ACT2*	*TUA2*	*PP2A*	*CYP19*	*DNAJ*	*TBP2*	*TUB3*	*HIS3*	*U2AF*	*TEF2*	*eIF-3A*	*eIF-3C*	*GAPDH*
GeNorm	*CYP19* | *TUA2*		*ACT2*	*DNAJ*	*PP2A*	*TBP2*	*TUB3*	*U2AF*	*TEF2*	*HIS3*	*eIF-3C*	*eIF-3A*	*GAPDH*
RefFinder	*ACT2*	*TUA2*	*CYP19*	*PP2A*	*DNAJ*	*TBP2*	*TUB3*	*eIF-3C*	*U2AF*	*TEF2*	*HIS3*	*eIF-3A*	*GAPDH*
Cold stress													
Delta Ct	*PP2A*	*ACT2*	*DNAJ*	*TBP2*	*TEF2*	*TUA2*	*CYP19*	*HIS3*	*U2AF*	*TUB3*	*eIF-3C*	*GAPDH*	*eIF-3A*
BestKeeper	*eIF-3C*	*TUB3*	*TBP2*	*DNAJ*	*ACT2*	*TUA2*	*TEF2*	*U2AF*	*PP2A*	*CYP19*	*HIS3*	*GAPDH*	*eIF-3A*
NormFinder	*PP2A*	*ACT2*	*DNAJ*	*HIS3*	*TEF2*	*TBP2*	*CYP19*	*U2AF*	*TUA2*	*TUB3*	*eIF-3C*	*GAPDH*	*eIF-3A*
GeNorm	*TEF2* | *U2AF*		*PP2A*	*DNAJ*	*TBP2*	*ACT2*	*TUA2*	*CYP19*	*HIS3*	*TUB3*	*eIF-3C*	*GAPDH*	*eIF-3A*
RefFinder	*PP2A*	*ACT2*	*DNAJ*	*TEF2*	*TBP2*	*U2AF*	*eIF-3C*	*TUB3*	*TUA2*	*HIS3*	*CYP19*	*GAPDH*	*eIF-3A*
Osmotic stress													
Delta Ct	*DNAJ*	*PP2A*	*ACT2*	*TUB3*	*CYP19*	*U2AF*	*TBP2*	*HIS3*	*TEF2*	*TUA2*	*eIF-3C*	*eIF-3A*	*GAPDH*
BestKeeper	*eIF-3C*	*TEF2*	*U2AF*	*DNAJ*	*TUB3*	*TBP2*	*PP2A*	*ACT2*	*HIS3*	*CYP19*	*TUA2*	*eIF-3A*	*GAPDH*
NormFinder	*ACT2*	*CYP19*	*HIS3*	*PP2A*	*TUB3*	*DNAJ*	*TBP2*	*TUA2*	*U2AF*	*TEF2*	*eIF-3C*	*eIF-3A*	*GAPDH*
GeNorm	*DNAJ* | *U2AF*		*TEF2*	*TUB3*	*TBP2*	*PP2A*	*ACT2*	*CYP19*	*HIS3*	*TUA2*	*eIF-3C*	*eIF-3A*	*GAPDH*
RefFinder	*DNAJ*	*U2AF*	*ACT2*	*PP2A*	*TUB3*	*TEF2*	*CYP19*	*eIF-3C*	*TBP2*	*HIS3*	*TUA2*	*eIF-3A*	*GAPDH*
All samples													
Delta Ct	*PP2A*	*TBP2*	*CYP19*	*TEF2*	*DNAJ*	*HIS3*	*TUA2*	*TUB3*	*ACT2*	*U2AF*	*eIF-3C*	*eIF-3A*	*GAPDH*
BestKeeper	*eIF-3C*	*DNAJ*	*U2AF*	*TBP2*	*TUB3*	*TEF2*	*PP2A*	*ACT2*	*CYP19*	*TUA2*	*HIS3*	*eIF-3A*	*GAPDH*
NormFinder	*PP2A*	*CYP19*	*TBP2*	*HIS3*	*TEF2*	*TUA2*	*DNAJ*	*TUB3*	*ACT2*	*U2AF*	*eIF-3C*	*eIF-3A*	*GAPDH*
GeNorm	*PP2A* | *TBP2*		*DNAJ*	*TEF2*	*TUB3*	*CYP19*	*TUA2*	*HIS3*	*ACT2*	*U2AF*	*eIF-3C*	*eIF-3A*	*GAPDH*
RefFinder	*PP2A*	*TBP2*	*DNAJ*	*CYP19*	*TEF2*	*eIF-3C*	*TUB3*	*HIS3*	*TUA2*	*U2AF*	*ACT2*	*eIF-3A*	*GAPDH*

Gene name	*ACT2*	*CYP19*	*DNAJ*	*eIF-3A*	*eIF-3C*	*GAPDH*	*HIS3*	*PP2A*	*TBP2*	*TEF2*	*TUA2*	*TUB3*	*U2AF*
Number of times the best gene appears	9	1	5	1	6	0	2	11	8	1	2	0	2

## Supplementary Materials

The following are available online at https://www.mdpi.com/2073-4425/10/6/451/s1. Figure S1: The top six most stable reference genes generated by delta Ct, BestKeeper, NormFinder, and geNorm. The purple, yellow, green, pink, and circles each contain the top six most stable reference genes of delta Ct, BestKeeper, NormFinder, and geNorm, respectively. The genes in the overlap area are the ones confirmed as the top six most stable reference genes by more than one algorithm. (A) Different genotypes, (B) different developmental stages, (C) different tissues, (D) salt stress, (E) heat stress, (F) cold stress, (G) osmotic stress, and (H) all samples. Figure S2: Expression stability of 13 candidate reference genes calculated by RefFinder. The lower Geomean value indicates a more stable expression. (A) Different genotypes, (B) different developmental stages, (C) different tissues, (D) salt stress, (E) heat stress, (F) cold stress, (G) osmotic stress, and (H) all samples.

## References

[1] Xie W.G., Zhang J.C., Zhao X.H., Zhang J.Q., Wang Y.R. (2015). Siberian wild rye (*Elymus sibiricus* L.): Genetic diversity of germplasm determined using DNA fingerprinting and SCoT markers. Biochem. Syst. Ecol..

[2] Zhou Q., Luo D., Ma L.C., Xie W.G., Wang Y., Wang Y.R., Liu Z.P. (2016). Development and cross-species transferability of EST-SSR markers in Siberian wildrye (*Elymus sibiricus* L.) using Illumina sequencing. Sci. Rep..

[3] Zhang Z.Y., Zhang J.C., Zhao X.H., Xie W.G., Wang Y.R. (2016). Assessing and broadening genetic diversity of *Elymus sibiricus* germplasm for the improvement of seed shattering. Molecules.

[4] Zhao X.H., Xie W.G., Zhang J.C., Zhang Z.Y., Wang Y.R. (2017). Histological characteristics, cell wall hydrolytic enzymes activity and candidate genes expression associated with seed shattering of *Elymus sibiricus* accessions. Front. Plant Sci..

[5] Wang M.Y., Hou L.Y., Zhu Y.Q., Zhang Q., Wang H., Xia F.S., Chen L.L., Mao P.S., Hannaway D.B. (2018). Siberian wildrye seed yield limited by assimilate source. Field Crops Res..

[6] Xie W.G., Zhao X.H., Zhang J.Q., Wang Y.R., Liu W.X. (2015). Assessment of genetic diversity of Siberian wild rye (*Elymus sibiricus* L.) germplasms with variation of seed shattering and implication for future genetic improvement. Biochem. Syst. Ecol..

[7] Xie W.G., Zhang J.C., Zhao X.H., Zhang Z.Y., Wang Y.R. (2017). Transcriptome profiling of *Elymus sibiricus*, an important forage grass in Qinghai-Tibet plateau, reveals novel insights into candidate genes that potentially connected to seed shattering. BMC Plant Biol..

[8] Tong Z.G., Gao Z.H., Wang F., Zhou J., Zhang Z. (2009). Selection of reliable reference genes for gene expression studies in peach using real-time PCR. BMC Mol. Biol..

[9] Luo M., Gao Z., Li H., Li Q., Zhang C.X., Xu W.P., Song S.R., Ma C., Wang S.P. (2018). Selection of reference genes for miRNA qRT-PCR under abiotic stress in grapevine. Sci. Rep..

[10] Narancio R., John U., Mason J., Spangenberg G. (2018). Selection of optimal reference genes for quantitative RT-PCR transcript abundance analysis in white clover (*Trifolium repens* L.). Funct. Plant Biol..

[11] Expósito-Rodríguez M., Borges A.A., Borges-Pérez A., Pérez J.A. (2008). Selection of internal control genes for quantitative real-time RT-PCR studies during tomato development process. BMC Plant Biol..

[12] Sheshadri S., Nishanth M., Yamine V., Simon B. (2018). Effect of Melatonin on the stability and expression of reference genes in *Catharanthus roseus*. Sci. Rep..

[13] Guénin S., Mauriat M., Pelloux J., Van Wuytswinkel O., Bellini C., Gutierrez L. (2009). Normalization of qRT-PCR data: The necessity of adopting a systematic, experimental conditions-specific, validation of references. J. Exp. Bot..

[14] Gutierrez L., Mauriat M., Guénin S., Pelloux J., Lefebvre J.F., Louvet R., Rusterucci C., Moritz T., Guerineau F., Bellini C. (2008). The lack of a systematic validation of reference genes: A serious pitfall undervalued in reverse transcription-polymerase chain reaction (RT-PCR) analysis in plants. Plant Biotechnol. J..

[15] Ozsolak F., Milos P.M. (2011). RNA sequencing: Advances, challenges and opportunities. Nat. Rev. Genet..

[16] Jain M. (2011). Next-generation sequencing technologies for gene expression profiling in plants. Brief. Funct. Genom..

[17] Sinha P., Saxena R.K., Singh V.K., Krishnamurthy L., Varshney R.K. (2015). Selection and validation of housekeeping genes as reference for gene expression studies in pigeonpea (*Cajanus cajan*) under heat and salt stress conditions. Front. Plant Sci..

[18] Bao W., Qu Y., Shan X., Wan Y. (2016). Screening and validation of housekeeping genes of the root and cotyledon of *Cunninghamia lanceolata* under abiotic stresses by using quantitative real-time PCR. Int. J. Mol. Sci..

[19] Jain M., Nijhawan A., Tyagi A.K., Khurana J.P. (2006). Validation of housekeeping genes as internal control for studying gene expression in rice by quantitative real-time PCR. Biochem. Biophys. Res. Commun..

[20] Glare E., Divjak M., Bailey M., Walters E. (2002). β-Actin and GAPDH housekeeping gene expression in asthmatic airways is variable and not suitable for normalising mRNA levels. Thorax.

[21] Gao M.M., Liu Y.P., Ma X., Shuai Q., Gai J.Y., Li Y. (2017). Evaluation of reference genes for normalization of gene expression using quantitative RT-PCR under aluminum, cadmium, and heat stresses in soybean. PLoS ONE.

[22] Thellin O., Zorzi W., Lakaye B., De Borman B., Coumans B., Hennen G., Grisar T., Igout A., Heinen E. (1999). Housekeeping genes as internal standards: Use and limits. J. Biotechnol..

[23] Zhou Z., Cong P.H., Tian Y., Zhu Y.M. (2017). Using RNA-seq data to select reference genes for normalizing gene expression in apple roots. PLoS ONE.

[24] Dheda K., Huggett J., Chang J., Kim L., Bustin S., Johnson M., Rook G., Zumla A. (2005). The implications of using an inappropriate reference gene for real-time reverse transcription PCR data normalization. Anal. Biochem..

[25] Sang J., Han X.J., Liu M.Y., Qiao G.R., Jiang J., Zhuo R.Y. (2013). Selection and validation of reference genes for real-time quantitative PCR in hyperaccumulating ecotype of *Sedum alfredii* under different heavy metals stresses. PLoS ONE.

[26] Karuppaiya P., Yan X.X., Liao W., Wu J., Chen F., Tang L. (2017). Identification and validation of superior reference gene for gene expression normalization via RT-qPCR in staminate and pistillate flowers of *Jatropha curcas*—A biodiesel plant. PLoS ONE.

[27] Xiao Z., Sun X.B., Liu X.Q., Li C., He L.S., Chen S.P., Su J.L. (2016). Selection of reliable reference genes for gene expression studies on *Rhododendron molle* G. Don. Front. Plant Sci..

[28] Wu Z.J., Tian C., Jiang Q., Li X.H., Zhuang J. (2016). Selection of suitable reference genes for qRT-PCR normalization during leaf development and hormonal stimuli in tea plant (*Camellia sinensis*). Sci. Rep..

[29] Wan Q., Chen S.L., Shan Z.H., Yang Z.L., Chen L.M., Zhang C.J., Yuan S.L., Hao Q.N., Zhang X.J., Qiu D.Z. (2017). Stability evaluation of reference genes for gene expression analysis by RT-qPCR in soybean under different conditions. PLoS ONE.

[30] Wang H.B., Wang J.J., Jiang J.F., Chen S.M., Guan Z.Y., Liao Y., Chen F.D. (2014). Reference genes for normalizing transcription in diploid and tetraploid *Arabidopsis*. Sci. Rep..

[31] Pabuayon I.M., Yamamoto N., Trinidad J.L., Longkumer T., Raorane M.L., Kohli A. (2016). Reference genes for accurate gene expression analyses across different tissues, developmental stages and genotypes in rice for drought tolerance. Rice.

[32] Cai J., Li P.F., Luo X., Chang T.L., Li J.X., Zhao Y.W., Xu Y. (2018). Selection of appropriate reference genes for the detection of rhythmic gene expression via quantitative real-time PCR in Tibetan hulless barley. PLoS ONE.

[33] Huang L.K., Yan H.D., Jiang X.M., Zhang Y., Zhang X.Q., Ji Y., Zeng B., Xu B., Yin G.H., Lee S. (2014). Reference gene selection for quantitative real-time reverse-transcriptase PCR in orchardgrass subjected to various abiotic stresses. Gene.

[34] Liu Y., Liu J., Xu L., Lai H., Chen Y., Yang Z.M., Huang B.R. (2017). Identification and validation of reference genes for seashore paspalum response to abiotic stresses. Int. J. Mol. Sci..

[35] Niu K.J., Shi Y., Ma H.L. (2017). Selection of candidate reference genes for gene expression analysis in Kentucky Bluegrass (*Poa pratensis* L.) under abiotic stress. Front. Plant Sci..

[36] Nguyen D.Q., Eamens A.L., Grof C.P. (2018). Reference gene identification for reliable normalisation of quantitative RT-PCR data in *Setaria viridis*. Plant Methods.

[37] Zhao X.H., Jiang X., Zhao K., Zhao X.H., Yin J., Xie W.G. (2015). Screening of germplasm with low seed shattering rate and evaluation on agronomic traits in *Elymus sibiricus* L. (Chinese with English abstract). J. Plant Genet. Resour..

[38] Vandesompele J., De Preter K., Pattyn F., Poppe B., Van Roy N., De Paepe A., Speleman F. (2002). Accurate normalization of real-time quantitative RT-PCR data by geometric averaging of multiple internal control genes. Genome Biol..

[39] Andersen C.L., Jensen J.L., Ørntoft T.F. (2004). Normalization of real-time quantitative reverse transcription-PCR data: A model-based variance estimation approach to identify genes suited for normalization, applied to bladder and colon cancer data sets. Cancer Res..

[40] Pfaffl M.W., Tichopad A., Prgomet C., Neuvians T.P. (2004). Determination of stable housekeeping genes, differentially regulated target genes and sample integrity: BestKeeper–Excel-based tool using pair-wise correlations. Biotechnol. Lett..

[41] Silver N., Best S., Jiang J., Thein S.L. (2006). Selection of housekeeping genes for gene expression studies in human reticulocytes using real-time PCR. BMC Mol. Biol..

[42] Xie F.L., Xiao P., Chen D.L., Xu L., Zhang B.H. (2012). miRDeepFinder: A miRNA analysis tool for deep sequencing of plant small RNAs. Plant Mol. Biol..

[43] Chen Y., Tan Z.Q., Hu B.Y., Yang Z.M., Xu B., Zhuang L.L., Huang B.R. (2015). Selection and validation of reference genes for target gene analysis with quantitative RT-PCR in leaves and roots of bermudagrass under four different abiotic stresses. Physiol. Plant..

[44] Radonić A., Thulke S., Mackay I.M., Landt O., Siegert W., Nitsche A. (2004). Guideline to reference gene selection for quantitative real-time PCR. Biochem. Biophys. Res. Commun..

[45] Livak K.J., Schmittgen T.D. (2001). Analysis of relative gene expression data using real-time quantitative PCR and the 2^− ΔΔCT^ method. Methods.

[46] Silveira É.D., Alves-Ferreira M., Guimarães L.A., da Silva F.R., de Campos Carneiro V.T. (2009). Selection of reference genes for quantitative real-time PCR expression studies in the apomictic and sexual grass *Brachiaria brizantha*. BMC Plant Biol..

[47] Udvardi M.K., Czechowski T., Scheible W.-R. (2008). Eleven golden rules of quantitative RT-PCR. Plant Cell.

[48] Fei X.T., Shi Q.Q., Yang T.X., Fei Z.X., Wei A.Z. (2018). Expression stabilities of ten candidate reference genes for RT-qPCR in *Zanthoxylum bungeanum* Maxim. Molecules.

[49] Xiang Q.J., Li J., Qin P., He M.L., Yu X.M., Zhao K., Zhang X.P., Ma M.G., Chen Q., Chen X.Q. (2018). Identification and evaluation of reference genes for qRT-PCR studies in *Lentinula edodes*. PLoS ONE.

[50] Dai F.W., Zhao X.T., Tang C.M., Wang Z.J., Kuang Z.S., Li Z.Y., Huang J., Luo G.Q. (2018). Identification and validation of reference genes for qRT-PCR analysis in mulberry (*Morus alba* L.). PLoS ONE.

[51] Zheng T.C., Chen Z.L., Ju Y.Q., Zhang H., Cai M., Pan H.T., Zhang Q.X. (2018). Reference gene selection for qRT-PCR analysis of flower development in *Lagerstroemia indica* and *L. speciosa*. PLoS ONE.

[52] Chen J.C., Huang Z.F., Huang H.J., Wei S.H., Liu Y., Jiang C.L., Zhang J., Zhang C.X. (2017). Selection of relatively exact reference genes for gene expression studies in goosegrass (*Eleusine indica*) under herbicide stress. Sci. Rep..

[53] Duan M.M., Wang J.L., Zhang X.H., Yang H.H., Wang H.P., Qiu Y., Song J.P., Guo Y.D., Li X.X. (2017). Identification of optimal reference genes for expression analysis in Radish (*Raphanus sativus* L.) and its relatives based on expression stability. Front. Plant Sci..

[54] Wang X., Ma X., Huang L.K., Zhang X.Q. (2015). Identification of the valid reference genes for quantitative RT-PCR in annual ryegrass (*Lolium multiflorum*) under salt stress. Molecules.

[55] Huang L.K., Yan H.D., Jiang X.M., Zhang X.Q., Zhang Y.W., Huang X., Zhang Y., Miao J.M., Xu B., Frazier T. (2014). Evaluation of candidate reference genes for normalization of quantitative RT-PCR in switchgrass under various abiotic stress conditions. BioEnergy Res..

[56] Kesten C., Menna A., Sanchez-Rodriguez C. (2017). Regulation of cellulose synthesis in response to stress. Curr. Opin. Plant Biol..

[57] Wang L.Q., Guo K., Li Y., Tu Y.Y., Hu H.Z., Wang B.R., Cui X.C., Peng L.C. (2010). Expression profiling and integrative analysis of the *CESA*/*CSL* superfamily in rice. BMC Plant Biol..

[58] Hazen S.P., Scott-Craig J.S., Walton J.D. (2002). Cellulose synthase-like genes of rice. Plant Physiol..

[59] Nakano T., Kimbara J., Fujisawa M., Kitagawa M., Ihashi N., Maeda H., Kasumi T., Ito Y. (2012). MACROCALYX and JOINTLESS interact in the transcriptional regulation of tomato fruit abscission zone development. Plant Physiol..

[60] Nunes A., Delatorre C., Merotto A. (2014). Gene expression related to seed shattering and the cell wall in cultivated and weedy rice. Plant Biol..

[61] Li Y.H., Cheng X.J., Fu Y.Q., Wu Q.Q., Guo Y.L., Peng J.Y., Zhang W., He B. (2018). A genome-wide analysis of the *cellulose synthase-like* (*Csl*) gene family in maize (*Zea mays*). PeerJ Preprints.

[62] Hamann T., Osborne E., Youngs H.L., Misson J., Nussaume L., Somerville C. (2004). Global expression analysis of *CESA* and *CSL* genes in *Arabidopsis*. Cellulose.

[63] Wu J.Y., Zhang H.N., Liu L.Q., Li W.C., Wei Y.Z., Shi S.Y. (2016). Validation of reference genes for RT-qPCR studies of gene expression in preharvest and postharvest longan fruits under different experimental conditions. Front. Plant Sci..

[64] Caño-Delgado A., Penfield S., Smith C., Catley M., Bevan M. (2003). Reduced cellulose synthesis invokes lignification and defense responses in *Arabidopsis thaliana*. Plant J..

[65] Yoon J., Cho L.-H., Antt H.W., Koh H.-J., An G. (2017). KNOX protein OSH15 induces grain shattering by repressing lignin biosynthesis genes. Plant Physiol..

[66] Stolle-Smits T., Beekhuizen J.G., Kok M.T., Pijnenburg M., Recourt K., Derksen J., Voragen A.G. (1999). Changes in cell wall polysaccharides of green bean pods during development. Plant Physiol..

[67] Doblin M.S., Kurek I., Jacob-Wilk D., Delmer D.P. (2002). Cellulose biosynthesis in plants: From genes to rosettes. Plant Cell Physiol..

[68] Jithesh M., Shukla P.S., Kant P., Joshi J., Critchley A.T., Prithiviraj B. (2018). Physiological and transcriptomics analyses reveal that *Ascophyllum nodosum* extracts induce salinity tolerance in *Arabidopsis* by regulating the expression of stress responsive genes. J. Plant Growth Regul..

[69] Zhang J.L., Shi H.Z. (2013). Physiological and molecular mechanisms of plant salt tolerance. Photosynth. Res..

[70] Zheng M., Wang Y.H., Liu K., Shu H.M., Zhou Z.G. (2012). Protein expression changes during cotton fiber elongation in response to low temperature stress. J. Plant Physiol..

[71] Dametto A., Sperotto R.A., Adamski J.M., Blasi É.A., Cargnelutti D., de Oliveira L.F., Ricachenevsky F.K., Fregonezi J.N., Mariath J.E., da Cruz R.P. (2015). Cold tolerance in rice germinating seeds revealed by deep RNAseq analysis of contrasting *indica* genotypes. Plant Sci..

[72] Ko J.-H., Prassinos C., Keathley D., Han K.-H. (2011). Novel aspects of transcriptional regulation in the winter survival and maintenance mechanism of poplar. Tree Physiol..

[73] Chen J., Lv F.J., Liu J.R., Ma Y.N., Wang Y.H., Chen B.L., Meng Y.L., Zhou Z.G., Oosterhuis D.M. (2014). Effect of late planting and shading on cellulose synthesis during cotton fiber secondary wall development. PLoS ONE.

[74] Zhang F., Huang L.Y., Wang W.S., Zhao X.Q., Zhu L.H., Fu B.Y., Li Z.K. (2012). Genome-wide gene expression profiling of introgressed *indica* rice alleles associated with seedling cold tolerance improvement in a *japonica* rice background. BMC Genom..

