# Supplementary material for: Selection of Suitable Reference Genes for RT-qPCR Gene Expression Analysis in Siberian Wild Rye (Elymus sibiricus) under Different Experimental Conditions
Source: Genes (Basel). 2019 Jun 13;10(6):451. doi: 10.3390/genes10060451 (PMC6627066; doi:10.3390/genes10060451)
Supplement: Supplementary file 1 [file genes-10-00451-s001.pdf]

Table S1. Information of six *Elymus sibiricus* accessions used in this study.

Genotype	Full name	Origin	Longitude/°E	Latitude/°N	Altitude/m	Status
ZN	ZHN03	Zhuoni, Gansu, China	103.5692	34.5856	2605	Wild
XH	XH09	Xiahe, Gansu, China	102.5586	34.7528	3171	Wild
MQ	MQ01	Maqu, Gansu, China	102.1114	34	3475	Wild
HZ	HZ02	Hezuo, Gansu, China	102.9083	35.0614	2900	Wild
LQ	LQ03	Luqu, Gansu, China	102.6422	34.5511	3032	Wild
LT	LT04	Lintan, Gansu, China	103.6658	34.9192	2571	Wild

Table S2. Expression stability values of *E. sibiricus* potential reference genes calculated using NormFinder (The tabulated data of Figure 5).

Rank	Different genotypes	Different developmental stages	Different tissues	Salt stress	Heat stress	Cold stress	Drought stress	All samples
1	*TBP2* (0.10)	*PP2A* (0.11)	*TBP2* (0.07)	*ACT2* (0.15)	*ACT2* (0.13)	*PP2A* (0.23)	*ACT2* (0.22)	*PP2A* (0.29)
2	*eIF-3A* (0.14)	*TUA2* (0.20)	*CYP19* (0.08)	*PP2A* (0.23)	*TUA2* (0.19)	*ACT2* (0.43)	*CYP19* (0.28)	*TBP2* (0.45)
3	*TUA2* (0.15)	*TUB3* (0.31)	*eIF-3A* (0.12)	*TBP2* (0.24)	*PP2A* (0.23)	*DNAJ* (0.48)	*HIS3* (0.29)	*CYP19* (0.46)
4	*PP2A* (0.17)	*HIS3* (0.32)	*PP2A* (0.12)	*TUA2* (0.48)	*CYP19* (0.27)	*HIS3* (0.53)	*PP2A* (0.33)	*TEF2* (0.61)
5	*CYP19* (0.17)	*DNAJ* (0.35)	*TEF2* (0.22)	*HIS3* (0.58)	*DNAJ* (0.32)	*TEF2* (0.55)	*TUB3* (0.51)	*HIS3* (0.67)
6	*TEF2* (0.18)	*CYP19* (0.46)	*DNAJ* (0.45)	*CYP19* (0.78)	*TBP2* (0.50)	*TBP2* (0.56)	*DNAJ* (0.51)	*TUA2* (0.71)
7	*HIS3* (0.19)	*eIF-3C* (0.48)	*eIF-3C* (0.49)	*TUB3* (0.89)	*TUB3* (0.57)	*CYP19* (0.58)	*TBP2* (0.52)	*DNAJ* (0.74)
8	*ACT2* (0.21)	*ACT2* (0.48)	*HIS3* (0.52)	*TEF2* (0.95)	*HIS3* (0.68)	*U2AF* (0.62)	*TUA2* (0.55)	*TUB3* (0.79)
9	*DNAJ* (0.24)	*GAPDH* (0.53)	*GAPDH* (0.59)	*U2AF* (0.97)	*U2AF* (0.76)	*TUA2* (0.63)	*U2AF* (0.62)	*ACT2* (0.86)
10	*eIF-3C* (0.24)	*TEF2* (0.61)	*TUA2* (0.85)	*DNAJ* (1.16)	*TEF2* (0.84)	*TUB3* (0.87)	*TEF2* (0.75)	*U2AF* (1.40)
11	*TUB3* (0.24)	*eIF-3A* (0.69)	*U2AF* (1.09)	*eIF-3A* (1.19)	*eIF-3A* (1.27)	*eIF-3C* (1.14)	*eIF-3C* (1.44)	*eIF-3A* (1.57)
12	*GAPDH* (0.35)	*TBP2* (0.69)	*TUB3* (1.11)	*eIF-3C* (1.87)	*eIF-3C* (1.38)	*GAPDH* (1.43)	*eIF-3A* (1.55)	*eIF-3C* (1.59)
13	*U2AF* (0.78)	*U2AF* (0.90)	*ACT2* (2.38)	*GAPDH* (3.34)	*GAPDH* (1.55)	*eIF-3A* (1.77)	*GAPDH* (2.96)	*GAPDH* (1.96)

Table S3. Expression stability of candidate reference genes calculated by BestKeeper (The tabulated data of Figure 6).

Rank	Different genotypes	Different developmental stages	Different tissues	Salt stress	Heat stress	Cold stress	Drought stress	All samples
1	*HIS3*	*DNAJ*	*TBP2*	*eIF-3C*	*eIF-3C*	*eIF-3C*	*eIF-3C*	*eIF-3C*
SD	0.28	0.16	0.24	0.70	2.10	1.71	0.81	1.14
2	*ACT2*	*TUA2*	*DNAJ*	*DNAJ*	*TEF2*	*TUB3*	*TEF2*	*DNAJ*
SD	0.29	0.22	0.33	0.77	2.17	1.97	1.45	1.60
3	*CYP19*	*HIS3*	*PP2A*	*U2AF*	*U2AF*	*TBP2*	*U2AF*	*U2AF*
SD	0.37	0.27	0.38	1.10	2.41	2.07	1.60	1.80
4	*DNAJ*	*ACT2*	*eIF-3C*	*TUB3*	*TUB3*	*DNAJ*	*DNAJ*	*TBP2*
SD	0.38	0.31	0.48	1.13	2.52	2.49	1.73	1.85
5	*eIF-3C*	*CYP19*	*eIF-3A*	*TEF2*	*TBP2*	*ACT2*	*TUB3*	*TUB3*
SD	0.40	0.38	0.54	1.24	2.52	2.59	1.81	1.87
6	*TBP2*	*eIF-3C*	*CYP19*	*PP2A*	*PP2A*	*TEF2*	*TBP2*	*TEF2*
SD	0.41	0.42	0.55	1.61	2.95	2.66	1.81	2.06
7	*TUA2*	*PP2A*	*HIS3*	*TBP2*	*DNAJ*	*TUA2*	*PP2A*	*PP2A*
SD	0.41	0.43	0.61	1.83	2.98	2.66	2.06	2.24
8	*TEF2*	*TUB3*	*TEF2*	*ACT2*	*ACT2*	*U2AF*	*ACT2*	*ACT2*
SD	0.45	0.54	0.65	2.04	3.44	2.80	2.28	2.32
9	*TUB3*	*GAPDH*	*GAPDH*	*TUA2*	*TUA2*	*PP2A*	*HIS3*	*CYP19*
SD	0.47	0.73	0.71	2.70	3.46	2.88	2.53	2.55
10	*eIF-3A*	*TBP2*	*TUA2*	*HIS3*	*CYP19*	*CYP19*	*CYP19*	*TUA2*
SD	0.49	0.79	1.08	2.82	3.49	2.89	2.61	2.75
11	*PP2A*	*TEF2*	*U2AF*	*CYP19*	*HIS3*	*HIS3*	*TUA2*	*HIS3*
SD	0.49	0.99	1.18	3.01	3.99	3.33	2.88	2.99
12	*GAPDH*	*eIF-3A*	*TUB3*	*eIF-3A*	*eIF-3A*	*GAPDH*	*eIF-3A*	*eIF-3A*
SD	0.64	1.05	1.22	3.38	4.55	4.04	3.34	3.91
13	*U2AF*	*U2AF*	*ACT2*	*GAPDH*	*GAPDH*	*eIF-3A*	*GAPDH*	*GAPDH*
SD	0.94	1.08	2.36	5.36	4.62	4.51	5.09	4.04

Table S4. Expression stability values for 13 candidate reference genes calculated via Delta Ct (The tabulated data of Figure 7).

Rank	Different genotypes	Different developmental stages	Different tissues	Salt stress	Heat stress	Cold stress	Drought stress	All samples
1	*TBP2*	*PP2A*	*PP2A*	*ACT2*	*ACT2*	*PP2A*	*DNAJ*	*PP2A*
SD	0.40	0.76	1.03	1.55	1.11	1.23	1.36	1.43
2	*HIS3*	*TUA2*	*TBP2*	*PP2A*	*TUA2*	*ACT2*	*PP2A*	*TBP2*
SD	0.43	0.77	1.03	1.57	1.12	1.27	1.38	1.50
3	*CYP19*	*HIS3*	*eIF-3A*	*TBP2*	*PP2A*	*DNAJ*	*ACT2*	*CYP19*
SD	0.44	0.82	1.04	1.60	1.14	1.33	1.38	1.55
4	*eIF-3A*	*DNAJ*	*CYP19*	*TUA2*	*CYP19*	*TBP2*	*TUB3*	*TEF2*
SD	0.44	0.83	1.11	1.75	1.14	1.35	1.39	1.63
5	*ACT2*	*TUB3*	*TEF2*	*HIS3*	*DNAJ*	*TEF2*	*CYP19*	*DNAJ*
SD	0.45	0.85	1.15	1.80	1.16	1.36	1.45	1.66
6	*PP2A*	*CYP19*	*DNAJ*	*TUB3*	*TBP2*	*TUA2*	*U2AF*	*HIS3*
SD	0.45	0.93	1.19	1.88	1.19	1.38	1.45	1.67
7	*TUA2*	*ACT2*	*HIS3*	*U2AF*	*TUB3*	*CYP19*	*TBP2*	*TUA2*
SD	0.45	0.94	1.25	1.92	1.24	1.38	1.46	1.71
8	*TEF2*	*eIF-3C*	*GAPDH*	*CYP19*	*U2AF*	*HIS3*	*HIS3*	*TUB3*
SD	0.46	0.94	1.29	1.94	1.42	1.42	1.49	1.76
9	*DNAJ*	*GAPDH*	*eIF-3C*	*TEF2*	*HIS3*	*U2AF*	*TEF2*	*ACT2*
SD	0.49	1.03	1.33	1.94	1.43	1.43	1.55	1.83
10	*eIF-3C*	*TEF2*	*TUA2*	*DNAJ*	*TEF2*	*TUB3*	*TUA2*	*U2AF*
SD	0.49	1.12	1.56	2.05	1.51	1.60	1.62	1.86
11	*TUB3*	*TBP2*	*U2AF*	*eIF-3A*	*eIF-3A*	*eIF-3C*	*eIF-3C*	*eIF-3C*
SD	0.51	1.18	1.81	2.31	2.02	1.91	2.28	2.26
12	*GAPDH*	*eIF-3A*	*TUB3*	*eIF-3C*	*eIF-3C*	*GAPDH*	*eIF-3A*	*eIF-3A*
SD	0.61	1.2	1.85	2.84	2.11	2.29	2.62	2.55
13	*U2AF*	*U2AF*	*ACT2*	*GAPDH*	*GAPDH*	*eIF-3A*	*GAPDH*	*GAPDH*
SD	1.16	1.46	3.52	4.88	2.34	2.71	4.38	3.02

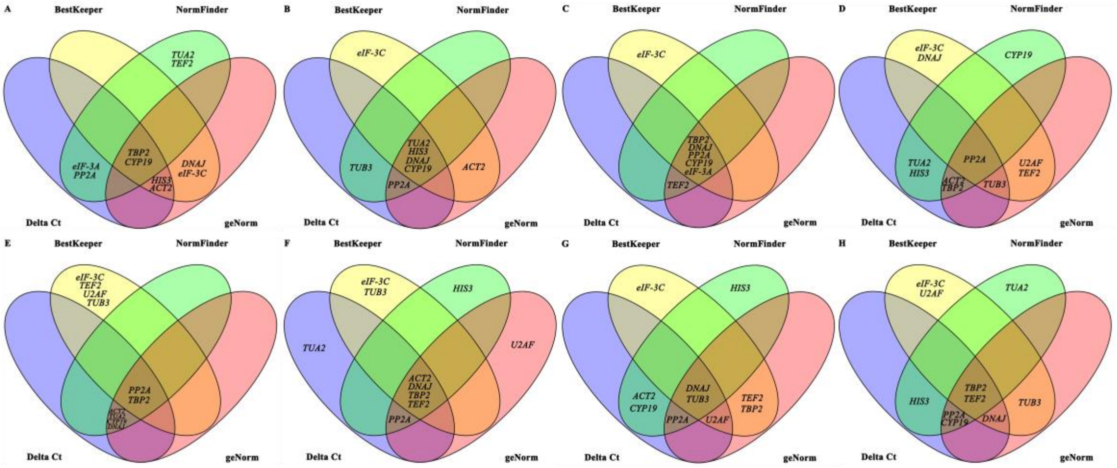

Figure S1

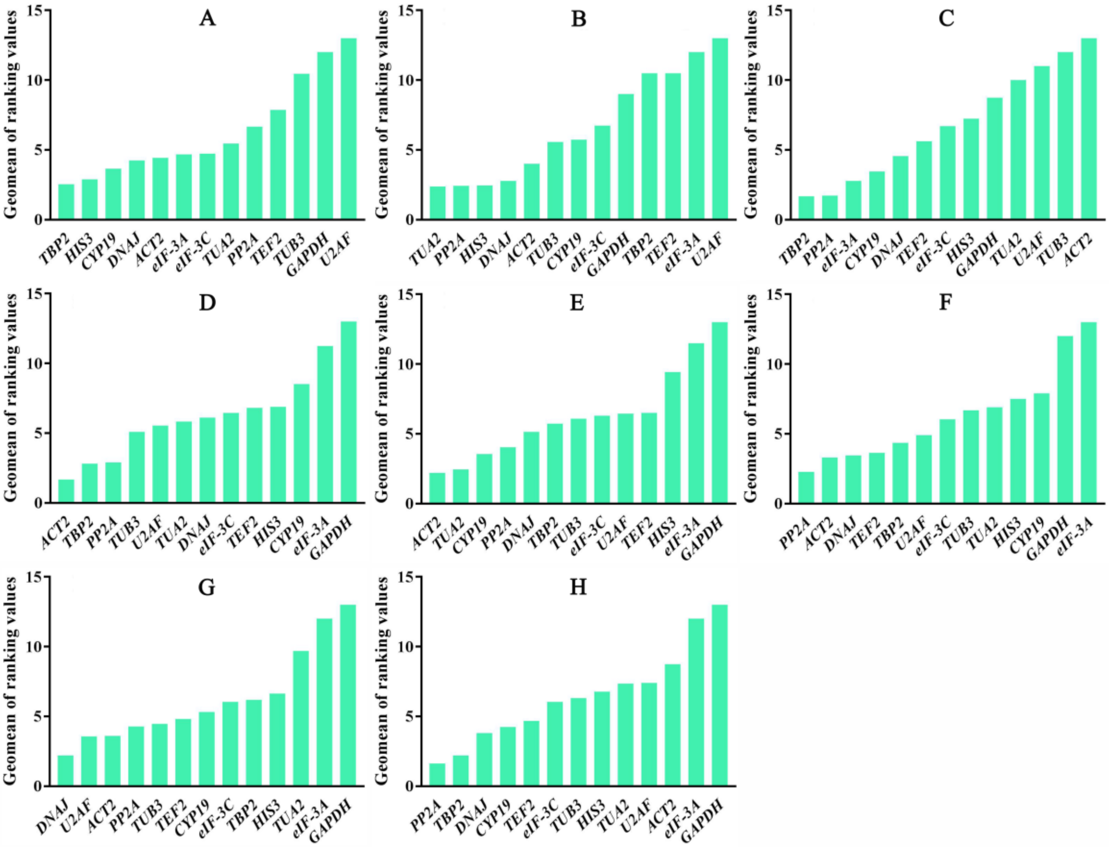

Figure S2
